# International opinion—The true cost of wounds for Canadians

**DOI:** 10.1111/iwj.14522

**Published:** 2023-12-12

**Authors:** Douglas Queen, Mariam Botros, Keith Harding

**Affiliations:** ^1^ Editor, IWJ; ^2^ CEO Wounds Canada Toronto Canada; ^3^ Editor‐in‐Chief, IWJ

## 1 INTRODUCTION

For many wound carers within Canada getting a handle on the costs associated with their management of chronic wounds is difficult, if not impossible. There are some published figures geographically, both national and provincial, but most of these are not “standardized” to permit comparison easily or directly.

One consistent theme from several international research studies, however,^1^, ^2^, ^3^, ^4^, ^5^, ^6^, ^7^, ^8^, ^9^, ^10^, ^11^, ^12^ is that they relate the costs of wounds, extrapolated or otherwise, to the total geographic healthcare costs. This provides a percentage figure as a benchmark, an approach which Canada has used previously.^13^

A simple literature search shows both the paucity of data generally across Canada, in some provinces and also the outdatedness of the data, with most of it being published over a decade ago. Due to the difficulties of capturing such cost data, most of these studies caution their results as an underestimate of the costs involved, with Canada being no different to the others.

A recent editorial in the International Wound Journal^14^ introduced an approach to estimate the possible costs of wound care using freely available governmental health data, population statistics, and the research findings of many international groups. Using these statistics and a simple formula provides an estimate of the likely costs of wound care within both Canada and the provinces and territories of which it is comprised.

## 2 METHODS

Using the methodology of Queen & Harding,^14^ the following formula was used to estimate the costs of wounds both in Canada and within its provinces and territories.

$$\text{EWCE}=\left[\text{PCHCS}\;x\;\mathrm{TP}\right]\;x\;\text{AWCCP}$$

where:

***EWCE**—Estimated Wound Care Expenditure (PPP International $)—our estimate of the likely wound care costs*.

***PCHCS**—*Per Capita *Health Care Spend (PPP International $)* ^15^, ^16^ *—current published* per capita *healthcare cost. For the purposes of this editorial, we focused on 2019 to eliminate and bias related to COVID‐19 costs. We will jump ahead to 2022 when they are published*.

***TP**—Total Population*.^17^

***AWCCP**—Average Wound Care Cost Percentage* ^1^, ^2^, ^3^, ^4^, ^5^, ^6^, ^7^, ^8^, ^9^, ^10^, ^11^, ^12^ *—several published studies have indicated that the percentage of total healthcare costs that is represented by the cost of wounds ranges from 2% on the low end to 5% on the high end. For the purposes of our calculations, remembering different geographies can be at differing evolutionary stages regarding wound care, we chose the median of 3.5% as the AWCCP*.

## 3 RESULTS

The following table provides a snapshot of the possible costs of wound care within Canada in the year 2022.

## 4 DISCUSSION

From the previous editorial,^14^ it was estimated that the costs of wound care in Canada were 6.9 billion PPP International Dollars (or 8.28 billion CAD—using the IMF Conversion Rate^18^). An international dollar is defined as being able to buy in the cited country a comparable amount of goods and services a U.S. dollar would buy in the United States.^19^

The 3 years between estimates were highly influenced by COVID which drove up per capita cost significantly during that period. This may or may not have artificially inflated the estimate for the cost of wounds. However, several studies showed that wound care was less than optimally delivered during this period and as such the costs of wound care would be higher it may be a real reflection of the true costs during the pandemic timeframe.^20^

The data presented in Table 1 provide a crucial estimate of the likely costs of wounds across Canada's provinces and territories. This comprehensive national perspective on wound costs significantly surpasses prior estimates from 2012.^13^ It acts as a vital benchmark at both provincial and national levels, serving as a tool for evaluating the effectiveness of standardizing wound care, advancing education, and training initiatives, and measuring the return on investment for government‐funded research and educational grants in this clinical field.^21^

**TABLE 1:** Estimated costs of wound care within Canada.

Province or Territory	Population (2022)	Per capita health spend 2022 (CAD$)	Estimated total health care spend 2022 (CAD$)	Estimated spend on wound care 2022 (CAD$)
Yukon	40 232	$15 884	$639 045 088	$22 366 578
Prince Edward Island	154 331	$8531	$1 316 597 761	$46 080 922
British Columbia	5 000 879	$8790	$43 957 726 410	$1 538 520 424
Ontario	14 223 942	$8213	$116 821 235 646	$4 088 743 248
Manitoba	1 342 153	$8417	$11 296 901 801	$395 391 563
Nova Scotia	969 383	$9536	$9 244 036 288	$323 541 270
Alberta	4 262 635	$8545	$36 424 216 075	$1 274 847 563
Quebec	8 501 833	$8701	$73 974 448 933	$2 589 105 713
New Brunswick	775 610	$8010	$6 212 636 100	$217 442 264
Saskatchewan	1 132 505	$5262	$5 959 241 310	$208 573 446
Nunavut	36 858	$21 978	$810 065 124	$28 352 279
Northwest Territories	41 070	$21 946	$901 322 220	$31 546 278
Newfoundland and Labrador	510 550	$9894	$5 051 381 700	$176 798 360
Canada	36 991 981	$8563	$316 762 333 303	$11 086 681 666

Identifying realistic estimates of the cost of wounds enables healthcare organizations to optimize resource allocation, facilitating the efficient allocation of budgets, and personnel to address the unique needs of patients. Secondly, this understanding acts as a catalyst for elevating the quality of care provided to individuals with wounds. Organizations are incentivized to invest in training, acquire necessary technology, and adopt best practices, all of which contribute to cost reduction while simultaneously enhancing patient outcomes.

Furthermore, comprehending the economic impact of wound care offers valuable insights to policymakers and healthcare leaders, shedding light on the broader economic implications of wound management. This knowledge serves as a foundation for informed decision‐making and the development of policies and research direction that support effective wound prevention and care practices.

While the IWJ has pledged to update the global picture annually, including Canada, we encourage national Canadian entities to provide the regional updates regularly to keep researchers up to date with the most recent estimates based on updated government statistics and any research findings.
